# Therapeutic responses and prognosis in Korean adult-onset Still’s disease

**DOI:** 10.1186/1479-5876-9-S2-P35

**Published:** 2011-11-23

**Authors:** Hyoun-Ah Kim, Jeong-Mi An, Chang-Hee Suh

**Affiliations:** 1 Dept. of Rheumatology, Ajou University School of Medicine, Suwon, Republic of Korea

## Background

To date, the treatment of adult onset Still’s disease (AOSD) has been largely empirical; therefore, this study was conducted to investigate the response to therapy and prognostic factors of AOSD. We describe the clinical features of 54 Korean patients with AOSD and provide an analysis of the therapeutic responses and prognostic factors associated with unfavorable outcomes and mortality in these cases.

## Method

Fifty-four Korean patients with AOSD were enrolled based on Yamaguchi’s criteria. We retrospectively analyzed the treatments and prognosis.

## Results

Thirty-nine patients (72.2%) were female and the average age at disease onset was 37.3 years. Twenty-nine patients had a monocyclic disease (53.7%), five had a polycyclic (9.3%) and fifteen had a chronic articular disease (27.7%) and five died (9.3%). The elevated ESR and corticosteroids refractoriness were associated with poor prognosis (*p*=0.023 and *p*=0.009, respectively). The patients that died were older than those survived (49.2 ± 11.8 vs 42.2 ± 14 year-old, *p*=0.024). Forty-two patients were treated with nonsteroidal anti-inflammatory drugs; however, they also needed corticosteroids and intravenous immunoglobulin (IVIG). Among 50 patients treated with high-dose corticosteroids, 21 patients (42%) were resistant to corticosteroids and treated with IVIG or anti-tumor necrosis factor (TNF) agents. Of the 23 patients medicated with IVIG, the prognosis was better in IVIG-responsive patients, indicating a therapeutic effect. Methotrexate was the most commonly used disease modifying antirheumatic drugs (27 patients, 50%) and the corticosteroid requirements were lower in the methotrexate-responsive patients.

## Conclusion

Approximately a half of Korean AOSD patients had a poor prognosis including polycyclic or chronic articular disease, or mortality. In addition, there was no clinical response to NSAIDs and 42% of patients were corticosteroids resistant and therefore required IVIG and anti-TNF agents. Methotrexate showed a corticosteroid-sparing effect. The elevated serum ESR and non-response to corticosteroids were significantly associated with poor prognosis. Finally, patients who died were older than those who survived.

